# Being certain about uncertainties: a robust evaluation method for high-dose-rate prostate brachytherapy treatment plans including the combination of uncertainties

**DOI:** 10.1007/s13246-023-01279-8

**Published:** 2023-05-30

**Authors:** Andrew C. Kennedy, Michael J. J. Douglass, Alexandre M. C. Santos

**Affiliations:** 1 School of Physical Sciences, University of Adelaide, Adelaide, SA 5005 Australia; 2 Department of Radiation Oncology, Royal Adelaide Hospital, Adelaide, SA 5000 Australia; 3 Australian Bragg Centre for Proton Therapy and Research, North Terrace, Adelaide, SA 5000 Australia

**Keywords:** Robust evaluation, Brachytherapy, Radiotherapy planning, Uncertainty

## Abstract

In high-dose-rate (HDR) prostate brachytherapy the combined effect of uncertainties cause a range of possible dose distributions deviating from the nominal plan, and which are not considered during treatment plan evaluation. This could lead to dosimetric misses for critical structures and overdosing of organs at risk. A robust evaluation method to assess the combination of uncertainties during plan evaluation is presented and demonstrated on one HDR prostate ultrasound treatment plan retrospectively. A range of uncertainty scenarios are simulated by changing six parameters in the nominal plan and calculating the corresponding dose distribution. Two methods are employed to change the parameters, a probabilistic approach using random number sampling to evaluate the likelihood of variation in dose distributions, and a combination of the most extreme possible values to access the worst-case dosimetric outcomes. One thousand probabilistic scenarios were run on the single treatment plan with 43.2% of scenarios passing seven of the eight clinical objectives. The prostate *D*_90_ had a standard deviation of 4.4%, with the worst case decreasing the dose by up to 27.2%. The urethra *D*_10_ was up to 29.3% higher than planned in the worst case. All DVH metrics in the probabilistic scenarios were found to be within acceptable clinical constraints for the plan under statistical tests for significance. The clinical significance of the results from the robust evaluation method presented on any individual treatment plan needs to be compared in the context of a historical data set that contains patient outcomes with robustness analysis data to ascertain a baseline acceptance.

## Introduction

High-Dose-Rate (HDR) Brachytherapy (BT) in the treatment of localized prostate cancer achieves higher cancer control rates than external beam therapy alone [1] due to superior dose escalation and a conformal dose distribution. This results in greater sparing of organs at risk (OAR) and coverage of the prostate target [2]. However, due to uncertainty contributions from the treatment planning process, intra-fractional motion, and the equipment used during treatment delivery, the nominal planned dose distribution will differ from the dose distribution delivered to the patient.

### Uncertainties

During treatment planning, inter- and intra-observer variations for delineating structures and the choice of imaging modality contribute to the overall uncertainty. Numerous studies have investigated the dosimetric impact of contouring variation. Rylander et al. [3] compared the lowest dose to the most irradiated 90% of the prostate target (*D*_90_) using trans-rectal ultrasound (TRUS) for contouring, and the same prostates contoured in magnetic resonance imaging (MRI), reporting a mean dose difference of 7% and standard deviation of 11% between the modalities. Contouring the rectum and urethra also has associated delineation uncertainties. While investigating the effect of interobserver contouring difference of the rectum, Chicas-Sett et al. [4] found the mean difference in dose to the most irradiated 1 cm^3^ of the volume was 3.9 ± 1.2%. Considerable research attention has been directed to needle reconstruction uncertainties, notably the needle tip [5–9]. Comparing 574 needles reconstructed on TRUS to cone-beam computer tomography (CBCT), Batchelar et al. [5] found 97% agreed within 3 mm. The TG-43 formalised [10, 11] dose calculation used by treatment planning systems (TPS) is based on several assumptions that contribute to the combined uncertainty. TG-43 is limited by the following: dose calculated in a homogeneous water phantom rather than patient-specific heterogeneous tissue; it does not account for the high Z material of the needles; scattering of radiation in and immediately around the patient is also not considered [12]. Ma et al. [13] found TG-43 overestimated *V*_100_ by 0.89% compared to Monte Carlo based calculations in HDR prostate BT. The source activity measured from 46 sources was calculated to have a root-mean-square difference of 3.3% (and as much as 13% difference) from the source manufacturer specifications [14].

The time elapsed between acquiring the image for treatment planning and the conclusion of irradiation is typically between 30 and 90 min [15] in US based planning. Significant movements in structures have been reported to occur during this interval [15]. Needle movements change the dose distribution delivered, and organ movements change the amount of dose received by the organ volumes, increasing overall uncertainty. Milickovic et al. [15] compared *D*_90_ due to movements in structures before and after irradiation using TRUS treatment planning for 25 patients finding a significant difference in prostate coverage (p < 0.001). Prostate oedema is of concern with permanent seed implant BT; however, in HDR-BT timeframes, it is said to be negligible [16], particularly within a single fraction.

The radiation source is driven on the end of a wire cable through each needle, stopping at planned dwell positions. The tension in the wire causes a positional uncertainty of 1 mm (*k* = 1) given by the manufacturers [17]. Source positioning precision has been documented to be as much as 2.3 mm [18]. As the source travels between dwell points, dose is delivered, called transit dose, which is not considered in the static dose distribution in the TPS. The dwell times are programmed to be reduced by the afterloader to compensate [19]; however, not accurately in all cases. Using Monte Carlo dose calculations, Fonseca et al. [20] found up to a 2.7% increase in dose from planned at points inside the prostate with time correction applied, primarily due to the entrance and exit of the source in each needle. The precision of dwell times has been measured by Johansen et al. [21], who calculated a variation of up to 0.4 s from planned.

In the worst cases, these individual uncertainty components can cause the failure of the treatment plan (TP) to meet the clinical objectives. Limited research has been conducted to assess the combined effect of uncertainty components on the dose delivered to individual patients, and the vast majority of work focuses on the effect of singular uncertainties on the dosimetric outcome. In a review of clinical brachytherapy uncertainties, Kirisits et al. [17] formulate an example uncertainty budget for a typical TRUS-based treatment planning procedure and stated achievable standards for each uncertainty component. While not the purpose of the uncertainty budget presented by Kirisits et al. [17], a logical utilization is in TP evaluation to quantify the impact of uncertainties on the dose distribution due to individual patient anatomy and plan parameters.

### Robust evaluation

Uncertainties are accounted for by increasing the irradiation target volume in each direction for the prostate, except posteriorly due to dose constraints to the rectum [17, 22]. Consequently, uncertainties are not directly considered during a patient’s TP evaluation. Increased margins result in increased dose to the soft tissue around the prostate and OAR. Robust evaluation is an alternative method to account for uncertainties by analysing a TP’s robustness to uncertainties. Robust evaluation is achieved using two approaches, the worst-case and probabilistic models [23]. In both cases, the dose distribution is recalculated for a number of combined uncertainties, with dose-volume-histogram (DVH) data stored for each iteration. This provides an uncertainty range for each DVH metric, which can be used to quantify the TPs’ robustness to uncertainties [24]. This additional information for plan evaluation could reduce target margins and increase the probability of target coverage and OAR sparing, hence improving treatment outcomes for the patient. Since margins accounting for uncertainty around the prostate are reduced when using the TRUS live planning technique, this becomes particularly important. Robust evaluation is used routinely in Intensity Modulated Proton Therapy (IMPT) [23, 25], and to a lesser degree in Intensity Modulated Radiotherapy (IMRT) [26] to evaluate TPs but is yet to be applied in HDR prostate BT. Robustness analysis in TP optimization is a current field of research in IMPT [27–29], IMRT [29], and BT [30–33]; using robust optimization to find a TP that is the most robust to uncertainties.

The present study aims to describe a framework for simulating uncertainty scenarios for a nominal TP in HDR prostate BT and calculating the possible range of DVH data. This will provide additional information for TP evaluation, and it is believed will result in the selection of TPs that have increased robustness to uncertainties.

## Method

A complete robust evaluation of a TP would involve the calculation of all possible dose distribution resulting from uncertainties, which is computationally unachievable due to the number of possible combinations of movements required to be simulated. A TP with 100 dwell points and dwell point movements simulated in six directions with only one moving at a time, and ignoring other changes to the plan, equates to over 6.5 × 10^77^ scenarios, making computation time unfeasible. To overcome this limitation in IMPT not all uncertainty scenarios are computed and are limited to scenarios of the most extreme patient setup and range uncertainties [34, 35]. The current study implements two robust evaluation approaches to overcome this limitation, probabilistic and worst-case. A treatment scenario is the TP generated for a single set of uncertainty values being used to make changes to the nominal plan [36]. The probabilistic approach uses a random number generator to generate uncertainty scenarios from known probability distributions and then calculates the dose distribution for that treatment scenario. Each probability distribution is sampled within a 90% confidence interval (CI), which is suggested by Korevaar et al. [23] since it is used in formulating CTV-PTV margins. The probabilistic scenarios will quantify the likely variation in dose distributions and hence the robustness of the TP to uncertainties. The worst-case approach simulates the extreme limits of the dose distributions that could occur, however unlikely. The values for changes to the nominal plan are combinations of the 90% CI limits for the probability distributions.

### Robust evaluation framework

**Fig. 1:**
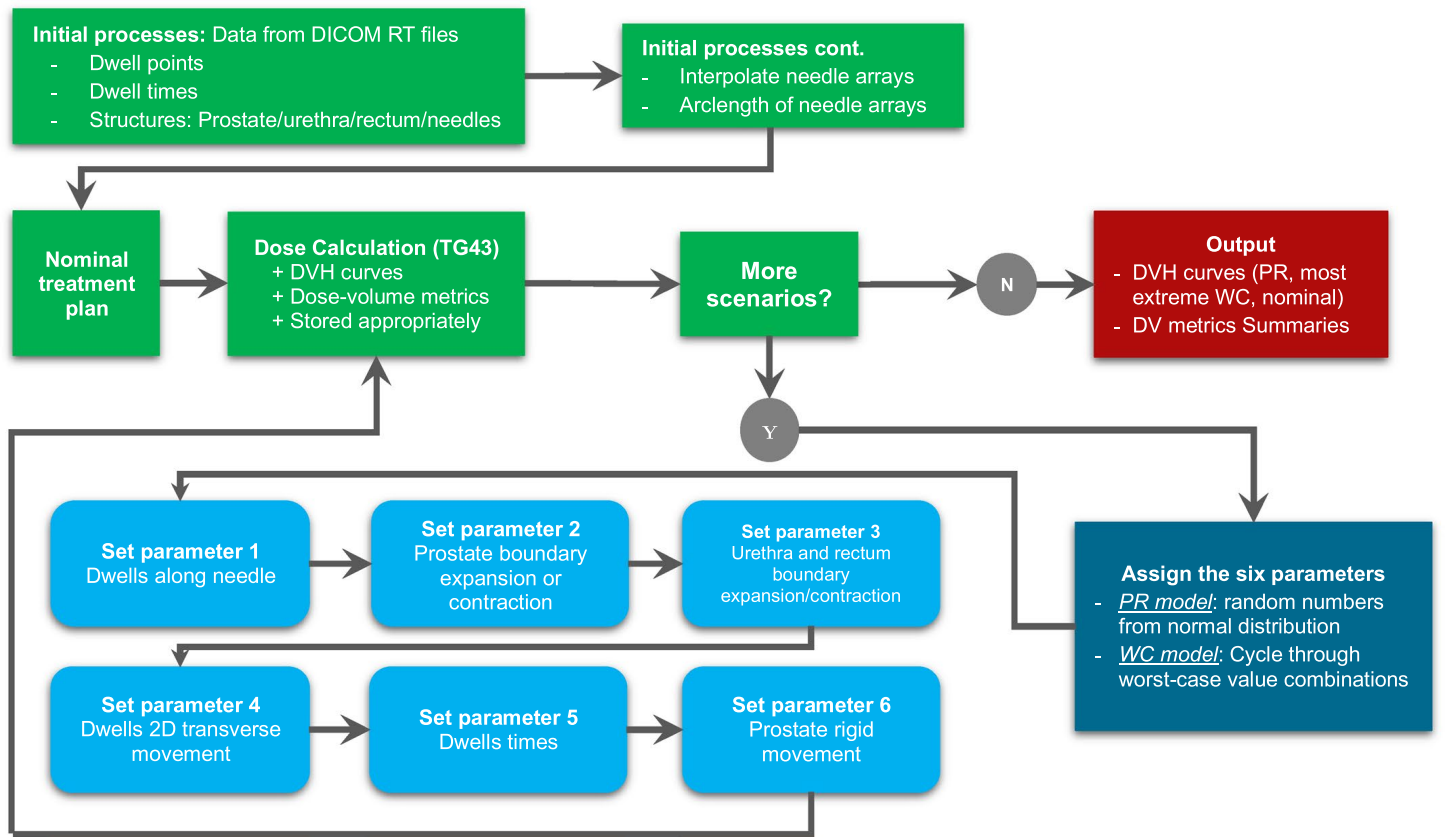
Robust evaluation algorithm flowchart for HDR prostate brachytherapy treatment plans to assess a plan’s robustness to uncertainties. *PR* probabilistic, *WC* worst-case

The process for robust evaluation in this paper is outlined below and shown in Fig. 1. It is adapted from the paper by Korevaar et al. [23].

1. Uncertainty components are combined into six common changes to the nominal TP (variable parameters).
2. TP variable parameters are sampled: probabilistic from an assumed normal distribution for each of the six variable parameters within a 90% CI and the worst-case from the set of 90% CI limit combinations from the same normal distributions used for the probabilistic scenarios.
3. The six variable parameters are used in making the changes to the nominal plan to form a treatment scenario, changing the structures or dwell times.
4. The dose distribution is calculated, and the DVH data is stored and added to the overall DVH plot that comprises all probabilistic scenarios, the nominal plan, and the most extreme worst-case scenarios. The DVH metrics are also stored.
5. Steps 2 to 4 are iterated until, for probabilistic, the pre-set number of scenarios are simulated so that the DVH metric means are in an acceptable CI; and for worst-case, the set of all combinations of worst-case uncertainty values are completed.
6. An overall plot of DVH curves (see Fig. 4), the mean (± 95% CI) for each DVH metric, and the number of plans which pass each DVH metric are presented for clinical plan suitability, see Table 1.

**Table 1:** DVH metrics with constraints that quantify the clinical objectives for the HDR prostate brachytherapy patient considered in this work

Prostate	Urethra (Gy)	Rectum
*D*_90_ ≥ 16 Gy	*D*_10_ ≤ 18.4	*V*_75_ ≤ 1.0 cc
*V*_100_ ≥ 90%	*D*_0.01 cc_ ≤ 18.4	*D*_0.1 cc_ ≤ 13 Gy
*V*_150_ ≤ 40%		
*V*_200_ ≤ 10%		

Step 6 is assessed against, and in the context of, historical robust evaluation treatment plans using the same method as outlined by Korevaar et al. [23], which is beyond the scope of the present framework of robust evaluation.

### Patient selection

**Fig. 2:**
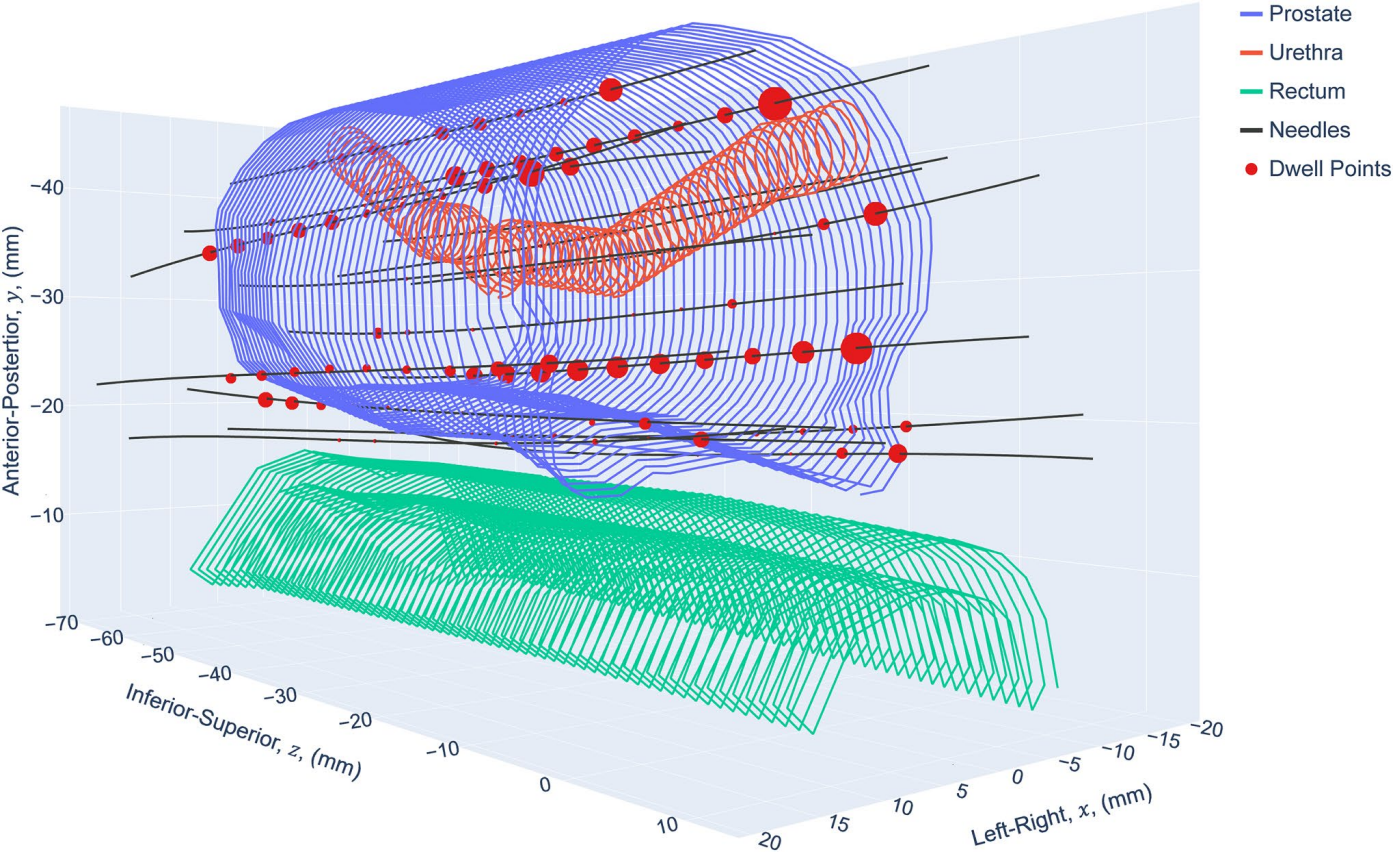
Patient geometry from the HDR prostate brachytherapy ultrasound treatment plan. The size of each red dwell point is directly proportionate to the relative dwell time for the source at that dwell point

A patient was selected retrospectively to demonstrate the robust evaluation approach developed. The patient’s plan was selected since it represents an average sized prostate volume and needle number for the patients treated at the clinic using the clinical objectives in Table 1. The plan was created with Vitesse v4.0.1 (Varian Medical Systems, Palo Alto, USA) and had a contoured prostate volume of 38.1 cc and 16 needles inserted; the geometry is shown in Fig. 2. An experienced radiation oncologist contoured all structures. The rectum was contoured 15 mm more inferiorly than the prostate, and the urethra 9 mm more inferiorly. Both the urethra and rectum were contoured 6 mm more superiorly.

### Uncertainties components

For this patient, the objective of the plan was to deliver 16 Gy as a single fraction boost with external beam radiotherapy. After needle insertion, a TRUS image was used to contour the prostate, urethra, and rectum, with the TRUS probe left in place until treatment was completed. The patient was under general anaesthesia and in the lithotomy position during the entire procedure. Uncertainty components in the technique were obtained from the literature (Table 2) and guided the range of simulated structural movements and dwell time changes, the variable parameters.

**Table 2:** Uncertainty magnitudes for HDR prostate brachytherapy treatments using live TRUS planning in a single fraction

Variable parameters	Uncertainty type	Published uncertainty values (mean ± SD | max)	Comments	Program input (standard deviations)
Parameter 1: Dwells move along the needle	Needle: Reconstruction SI (Needle Tip)	1.1 ± 0.1 mm | 8.0 mm [5]		Reconstruction: 1.1 mm Rigid: 0.34 mm Source: 1.0 mm Total: 1.5 mm
	Needle: Rigid movement SI	0.34 ± 0.12 mm | 0.58 mm^a^ [15]	SD approximated by 5th to 95th percentiles SI direction using needle-free length	
	Source positioning	0.2 ± 1.0 mm | 2.3 mm [18]		
Parameter 2: Prostate boundary expansion/contraction from geometric mean (prostate centre)	Prostate: Contouring	Width: 1.5 ± 1.3 mm | 6.1 mm [43] 1.6 ± 1.2 mm^a^ [50] Height: 1.8 ± 2.5 mm |18.2 mm [43] 1.7 ± 0.9 mm^a^ [50] Length: 2.5 ± 2.6 mm | 13.4 mm [43] 4.3 ± 2.2 mm^a^ [50]	Source [43] is from radiological studies	2.0 mm^b^
Parameter 3: Urethra boundary expanded/contracted in each transverse slice	Urethra: Contouring	Diameter: ± 0.5 mm		0.5 mm^b^
Parameter 3: Rectum boundary expanded/contracted in each transverse slice	Rectum: Contouring	Height and width: ± 1.0 mm		1.0 mm^b^
Parameter 4: Needle transverse plane movements PR: Random movement per needle WC prostate/urethra: Needles moveout from the prostate transverse centre line WC Rectum: all needles move posteriorly	Needle: Reconstruction transverse slice	Range: 0.5 to 4 mm [44] Median: 1.1 mm; Range: 0.0 to 9.6 mm [3] 0.2 ± 1.3 mm | 7.2 mm [51]	RL used since > PA [51] Not absolute values [51]	Reconstruction: 1.2 mm^b^ Rigid: 0.86 mm Total: 1.5 mm
	Needles: rigid movement transverse plane	0.86 ± 0.29 mm | 3.2 mm^a^ [15]	Mean 2D radial difference of 3 transverse planes. SD approximated by 5th to 95th percentiles	
Parameter 5: Dwell times percentage change	Dose Calculation	± 3.4% [52] ± 1% (Medium dosimetric corrections) [17] ± 3% (Treatment Planning) [17] ± 2% (Source Strength) [17] ± 3.3%; Range: − 13.0 to + 6% [14] ± 3% [17, 53] ± 3% | − 4.6% [54]	Best practices measured dose at 1 cm from source [52] Best practices, % relate to D_90_ and *V*_150_ of target, with adequate DVH binning [17]	Medium: 1% Treatment planning: 3% Source activity: 3% Total: 4.4%
	Source Activity		Difference between measured source strength and what the manufacturer stated [14] *k* = 1 SD given by the manufacturer [17] All source strength differences within 3% except one [54]	
Parameter 5: Dwell times add constant value	Source dwell timing	Dwell time precision: ± 0.06 s | 0.4 s [21]		0.06 s
Parameter 6: Prostate + Urethra 4-way movement	Prostate: rigid movement	AP: 0.5 ± 0.6 mm | 2.1 mm [15] LR: 0.1 ± 0.2 mm | 1.0 mm [15]		AP: 0.5 mm LR: 0.1 mm
	Urethra: rigid movement	0.9 ± 1.0 mm | 3.6 mm [15]	Mean 2D radial difference of 3 transverse planes [15]	
	Rectum: rigid movement	AP: 0.3 ± 0.4 mm | 1.4 mm [15] LR: 0.1 ± 0.2 mm | 0.7 mm [15]		

Uncertainty components are combined into six variable parameters on the basis that they result in the same change to the nominal plan. The last column forms the input to the robust evaluation program and are standard deviations that form six corresponding normal distributions for random sampling of each variable parameter, with the mean of each being zero. Worst-case values inputted into the program were ± 1.65 standard deviations, the 90% CIs. Directions are right-left (RL), superior-inferior (SI), and anterior–posterior (AP). *WC* worst-case scenario and *PR* probabilistic scenario. Absolute values are quoted unless otherwise commented

^a^Data extracted from figures or table

^b^Number-of-samples weighted average of means for absolute values and SD otherwise were used to calculate values

Inter-observer variations are generally more significant than intra-observer variations when contouring the prostate, and therefore, inter-observer uncertainty values were exclusively tabulated. Further, inter-modality differences due to using TRUS images for prostate contouring have shown not to be significant compared to the gold standard of MRI and are within inter-observer variations of MRI [3, 37–41].

To our knowledge, there has been no research into contour variations of the urethra or rectum using TRUS that include magnitude variations in transverse slices; however, one study has quantified variations in volume [42]. For the urethra, a radial uncertainty of ± 0.5 mm is tabulated since the structure is relatively small in each slice, and the rectum ± 1.0 mm due to its larger size. This correspondence between volume and one-dimensional distance variation in contours using TRUS can be seen in the classification of prostate volume and the length, width and height of the prostate recorded by Young et al. [43].

For needle reconstruction variations, studies involving phantom measurements are included [44] and intermodal differences in a patient data set [3]. Values are subdivided into needle-tip location variations, giving shifts in the superior-inferior direction and variations in the two-dimensional transverse plane. Transit dose is compensated by the afterloader to some degree and was not considered [19, 45].

The uncertainty values extracted from the literature were combined, in quadrature, into six variable parameters to operate on the nominal plan, forming a treatment scenario. Component uncertainties with multiple values obtained from literature were averaged, with higher weighting given to larger study sample sizes. The mean differences were used to quantify variation if the value was an absolute value; otherwise, the standard deviation (SD) was used if signed differences were stated.

### Scenario parameter setting

The algorithm that randomizes the six variable parameters in the TP is outlined in the following section and shown in the algorithm flowchart for robust evaluation in Fig. 1. After the changes have been made to the treatment files, the dose is calculated using the TG-43 dose calculation formalization [10, 11]. All simulations were run on an ASUS laptop with 16 GB of Ram and an Intel i7 6700HQ Processor.

#### Initial processes

In the initial processing of the TP for evaluation, the prostate, urethra, rectum, and needle structures are acquired from the radiotherapy treatment (RT) structures file, in DICOM format, into array structures; and the dwell points and times are obtained from the RT plan file. An array of approximately 6000 points is interpolated for each needle by Bezier curve fitting to the dwell points and needle end control. The cumulative distance of each interpolated point along each needle is obtained (the arc length array) and acts as a direct map between a needle array point and its distance from the needle tip by sharing the same position index in corresponding arrays. Below, ${\varvec{p}}$ stands for an array of points, ${\varvec{t}}$ an array of times, and ${\varvec{d}}$ an array of distances.

**INPUT:** RT plan and RT structure files

Dwell point array, ${\varvec{D}}$, where ${\varvec{p}}_{ij}^{D}$ is the $j$-th point in the $i$-th needle:

1 $${\varvec{D}} = {\varvec{p}}_{ij}^{D}$$

Dwell time array, ${\varvec{T}}$, where ${\varvec{t}}_{ij}^{ }$ is the $j$-th dwell time in the $i$-th needle:

2 $${\varvec{T}} = {\varvec{t}}_{ij}^{ }$$

Interpolated needle array, ${\varvec{N}}$, where ${\varvec{p}}_{ik}^{N}$ is the $k$-th point in the $i$-th needle:

3 $${\varvec{N}} = {\varvec{p}}_{ik}^{N}$$

Arclength array, ${\varvec{A}}$, where $d_{ik}^{ }$ is the $k$-th distance along the $i$-th needle of ${\varvec{p}}_{ik}^{N}$ from the needle tip:

4 $${\varvec{A}} = {\varvec{d}}_{ik}^{ }$$

Structures array, ${\varvec{S}}$, where ${\varvec{p}}_{mnl}^{S}$ is the *l*-th point in the *n*-th transverse slice of the $m$-th structure (prostate, urethra, rectum):

5 $${\varvec{S}} = {\varvec{p}}_{mnl}^{S}$$

**OUTPUT:** $${\varvec{D}},\user2{ N},\user2{ A},\user2{ S},{\varvec{T}}$$

#### Set parameter 1: Dwells move along the needle

Dwells are moved along the needle to simulate needle rigid movement, source positioning uncertainties during irradiation, and uncertainty in locating the needle tip in the image during treatment planning. The movement is made by using the map between interpolated needle points and the corresponding arc length array.

**INPUT:** $x =$ *Movement magnitude*

For each $i \in {\varvec{D}}_{ } , {\varvec{N}}_{ }$**:** {each needle}

For each $j \in {\varvec{D}}_{i}$**:** {each dwell point in $i$-th needle}

Find the index $k$ of the point in ${\varvec{N}}_{i}$ closest to the $j$-th dwell point in ${\varvec{D}}_{i}$:

6 $$k_{min} = k, \; s.t. \; \min \left( \parallel{{\varvec{p}}_{ij}^{D} - {\varvec{p}}_{ik}^{N}\parallel \; \forall\: k \in {\varvec{N}}_{i} } \right)$$

Now, find the index $k$ in ${\varvec{A}}_{i}$ that is closest to the new distance, ${\varvec{d}}_{{ik_{min} }}^{ } + x$:

7 $$k_{min,x} = k, \; s.t. \; \min \left( \parallel {{\varvec{d}}_{ik}^{ } - \left[ {{\varvec{d}}_{{ik_{min} }}^{ } + x} \right] \parallel \; \forall \: k \in {\varvec{A}}_{i} } \right)$$

Use the index $k_{min,x}$ in ${\varvec{N}}_{i}$ to obtain the new $j$-th dwell point shifted by $x$:

8 $${\varvec{p}}_{ij}^{D} \leftarrow {\varvec{p}}_{{ik_{min,x} }}^{N}$$

**OUTPUT:** updated $${\varvec{D}} = {\varvec{p}}_{ij}^{D}$$

#### Set parameter 2: prostate boundary expansion/contraction

All points on the prostate contour move with the same magnitude in the direction of the line joining a boundary point with the geometric centre of the prostate. This results in slice width inconsistencies, and resampling of the prostate contour is necessary at the original slice width.

**INPUT:** $x =$ *Movement magnitude*

Change distance between all points ${\varvec{p}}_{0nr}^{S}$ and the prostate (${\varvec{S}}_{0} )$ center, ${\text{mean}}\left( {{\varvec{p}}_{0nl}^{S} } \right)$:

9 $${\varvec{p}}_{0nl}^{S} \leftarrow \frac{{{\varvec{p}}_{0nl}^{S} - {\text{mean}}\left( {{\varvec{p}}_{0nl}^{S} } \right)}}{\parallel{{\varvec{p}}_{0nl}^{S} - {\text{mean}}\left( {{\varvec{p}}_{0nl}^{S} } \right)}\parallel} \times x + {\varvec{p}}_{0nl}^{S} \;\; \forall \: n,l \in {\varvec{S}}_{0}$$

Resample Structure to original slice widths.

Swap dimensions (slice $\leftrightarrow$ points):

10 $${\varvec{p}}_{0nl}^{S} \leftarrow {\varvec{p}}_{0ln}^{S}$$

Now, each $r$ contains the closest point from each original transverse slice and are curves along the superior-inferior (Z) direction instead of closed polygons in each transverse slice.

Interpolate along each curve defined by index $r$, increasing the number of points in $n$ to $q$ number of points:

For each $$l \in {\varvec{S}}_{0}$$**:**

11 $${\varvec{p}}_{pq}^{ } \leftarrow \text{Bspline}\left( {{\varvec{p}}_{0ln}^{S} } \right) \; \forall \: n \in {\varvec{S}}_{0}$$

Original slice width, with $Z$ being the set of z-coordinates of ${\varvec{p}}_{0nl}^{S}$**:**

12 $$y = \frac{\max \left( Z \right) - \min \left( Z \right)}{{{\text{total}}\left( n \right) - 1}}$$

Select points in ${\varvec{p}}_{pq}^{ }$ that correspond to the original slice width on either side of the mean z-coordinate of ${\varvec{p}}_{0nl}^{S}$, $\overline{Z}$:

13 $${\varvec{p}}_{0ln}^{S} \leftarrow {\varvec{p}}_{ly}^{ }, \;\;{\text{where}}\,y = {\text{subset of}} \; {\varvec{p}}_{pq}^{ }\; s.t. \left\{ { \ldots , \overline{Z} - 3y, \overline{Z} - 2y, \overline{Z} - y, \overline{Z}, \overline{Z} + y, \overline{Z} + 2y, \overline{Z} + 3y, \ldots } \right\}$$

Swap dimensions back (points $\leftrightarrow$ slice):

14 $${\varvec{p}}_{0ln}^{S} \leftarrow {\varvec{p}}_{0nl}^{S}$$

**OUTPUT:** updated $${\varvec{S}}_{0} = {\varvec{p}}_{0nl}^{S}$$

#### Set parameter 3: urethra and rectum boundary expansion/contraction

A uniform change is applied to 2-dimensional contours per slice, as the structures extend beyond the contoured inferior-superior region. The movement is the same magnitude and along the line joining the geometric centre of the structure in each slice to each boundary point.

**INPUT:** $x =$ *Movement magnitude;* $m = 1$ for urethra and $2$ for rectum.

For each $n \in {\varvec{S}}_{m}$**:** {slice $n$ held constant each iteration}

Change distance between all points in slice $n$ and the center of slice $n$, $\text{mean}\left( {{\varvec{p}}_{mnl}^{S} } \right)$, a distance ${\varvec{x}}$:

15 $${\varvec{p}}_{mnl}^{S} \leftarrow \frac{{{\varvec{p}}_{mnl}^{S} - \text{mean}\left( {{\varvec{p}}_{mnl}^{S} } \right)}}{\parallel{{\varvec{p}}_{mnl}^{S} - \text{mean}\left( {{\varvec{p}}_{mnl}^{S} } \right)}\parallel} \times x + {\varvec{p}}_{mnl}^{S} \;\;\forall \: l \in {\varvec{S}}_{0}$$

**OUTPUT:** updated $${\varvec{S}}_{{\varvec{m}}} = {\varvec{p}}_{mnl}^{S}$$

#### Set parameter 4: Dwells 2D transverse movement

Dwell points move within the transverse slice, which simulates variation in the reconstruction of the needles and rigid movement of the needle. The same movement magnitude is applied per needle.

**INPUT:** ${\varvec{x}} = {\varvec{x}}_{i} = \left[ {X,Y} \right]_{i}$ = *Movement vector, where* $X$ *is the left–right direction, and* $Y$ *is the anterior–posterior direction.*

For $${\varvec{i}} \in {\varvec{D}}:$$

16 $${\varvec{p}}_{ij}^{D} \leftarrow {\varvec{p}}_{ij}^{D} + {\varvec{x}}_{{\varvec{i}}} \user2{ } \; \; \forall \: j \in {\varvec{D}}_{{\varvec{i}}}$$

**OUTPUT:** updated $${\varvec{D}} = {\varvec{p}}_{ij}^{D}$$

#### Set parameter 5: Dwells times

The dose and source activity are proportional to dwell time, so dose calculation corrections were simulated by increasing or decreasing dwell times by a percentage. The precision in source dwell time is simulated by adding an amount to all dwell times.

**INPUT:** $x =$ *Time % change;* ${\varvec{y}} = {\varvec{y}}_{{{\varvec{ij}}}} =$ *Time constant change*

For each $i \in {\varvec{D}}_{ }$**:** {each needle}

For each $j \in {\varvec{D}}_{i}$**:** {each dwell point in $i$-th needle}

17 $${\varvec{t}}_{ij}^{ } \leftarrow {\varvec{t}}_{ij}^{ } \times x + y_{ij} \user2{ }$$

**OUTPUT:** updated $${\varvec{T}} = {\varvec{t}}_{ij}^{ }$$

#### Set parameter 6: prostate rigid movement

The rigid movement of the prostate and the rectum is simulated by moving only the prostate. The urethra and dwell points are also moved with the same magnitude and direction.

**INPUT:** ${\varvec{x}} = \left[ {X,Y,Z} \right]$ $=$ *Movement Vector*

Move prostate:

18 $${\varvec{p}}_{0nl}^{S} \leftarrow {\varvec{x}} + {\varvec{p}}_{0nl}^{S} \; \; \forall\: n,l \in {\varvec{S}}_{0}$$

Consequently, move urethra and dwell points:

19 $${\varvec{p}}_{1nl}^{S} \leftarrow {\varvec{x}} + {\varvec{p}}_{1nl}^{S} \; \; \forall \: n,l \in {\varvec{S}}_{1}$$

20 $${\varvec{p}}_{ij}^{D} \leftarrow {\varvec{p}}_{ij}^{D} + \user2{x } \;\; \forall \: i,j \in {\varvec{D}}_{ }$$

**OUTPUT:** updated $${\varvec{S}}_{0} = {\varvec{p}}_{0nl}^{S}$$, $${\varvec{S}}_{1} = {\varvec{p}}_{1nl}^{S}$$, $${\varvec{D}} = {\varvec{p}}_{ij}^{D}$$

### Statistical methods

Statistical significance was determined by a one-sample one-tail student* t*-test with a *p*-value less than 0.05 considered statistically significant. DVH metric distribution skewness was calculated using the Fisher-Pearson coefficient of skewness. For skewed distributions, the bootstrap percentile method [46] was utilized to compute the 95th percentile confidence interval of the median.

## Results

### Simulation timing

A total of 1000 probabilistic scenarios were run and 1944 worst-case scenarios. The mean time for a scenario, including changes to the nominal plan, obtaining dose points, the dose calculation and DVH construction, was 4.43 ± 0.67 s (mean ± SD) from the 2944 scenarios (probabilistic + worst-case). The dose calculation was the largest contributor taking on average 2.81 ± 0.53 s, and changes to the plan (setting variable parameters 1 to 6) had a processing time of 0.77 ± 0.35 s.

### Dose-volume data

For DVH metrics, 7.5% of scenarios passed all eight dose objectives from the probabilistic scenarios. The *V*_*200*_ constraint was allowed to slightly exceed the clinical objective for this patient’s anatomy; and, omitting this DVH metric increases the probabilistic pass-rate to 43.2%. Out of all scenarios (PR + worst-case), the maximum dose limit to the rectum, *D*_0.1 cc_, was met in 49.7% of scenarios. The maximum urethral dose, *D*_0.01 cc_, was acceptable in 56.1% of scenarios (probabilistic + worst-case), while for the prostate, *V*_100_ and *D*_90_ were met in 51.1% of scenarios (probabilistic + worst-case). Hotspots in the prostate were inconsistent between *V*_150_ and *V*_200_, with 97.2% and 46.8% of scenarios (probabilistic + worst-case) passing both constraints, respectively.

**Fig. 3:**
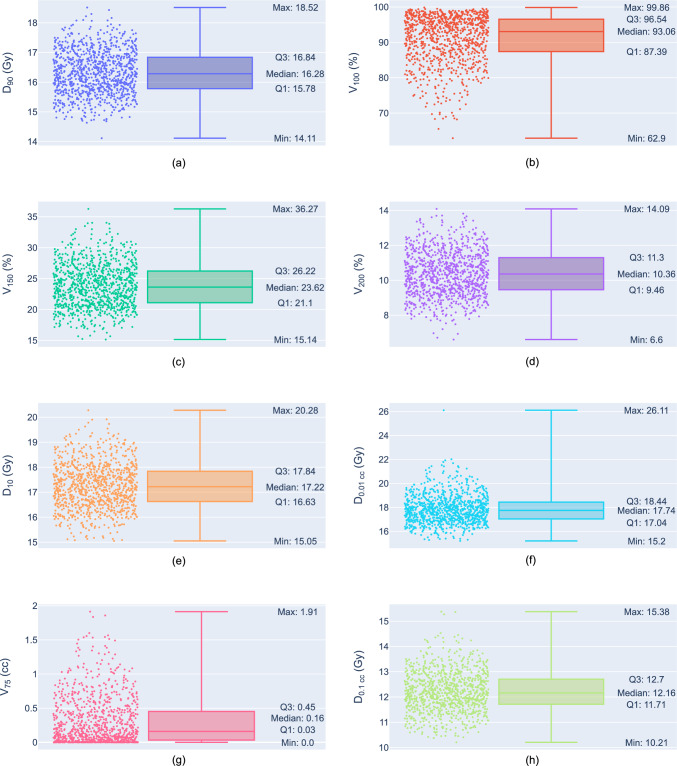
Distributions of DVH metrics as points and box plots for 1000 probabilistic uncertainty scenarios for a HDR prostate brachytherapy treatment plan. Prostate: **a** *D*_90_, **b** *V*_100_, **c** *V*_150_, **d** *V*_200_; urethra: **e** *D*_10_, **f** *D*_0.01 cc_; rectum: **g** *V*_75_, and **h** *D*_0.1 cc_. Distribution of points for *D*_90_, *V*_150_, *V*_200_, *D*_10_, *D*_0.01 cc_, *D*_0.1 cc_ approximate normal distributions

For the prostate, the *V*_100_ metric has an upper limit of 100% of the prostate volume, and since the target constraint is close to this upper limit (> 90%), the shape of the distribution for probabilistic simulation was negatively skewed (skewness = − 1.17). Similarly, the rectum DVH metric, *V*_75_, was positively skewed (skewness = 1.43) due to the nominal value being close to 0 cc. For both, the medians were used for central tendency accordingly, and a sample size of 1000 was used to compute the 95th percentile CIs of the median. The remaining six DVH metrics were appropriately close to approximating a normal distribution, as seen in Fig. 3.

**Fig. 4:**
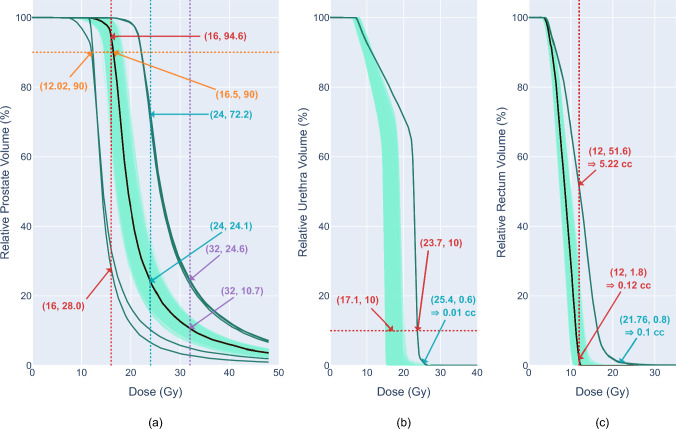
Dose-Volume Histogram’s: **a** prostate, **b** urethra, and **c** rectum. The nominal DVH plan is shown as a black line, the probabilistic scenarios are light green, and the dark green lines are the most extreme worst-case scenarios. DVH metric constraints are shown as dotted lines with interception points indicated. The maximum dose limits for the urethra and rectum worst cases are shown in light blue

DVH metrics are summarised for probabilistic simulations in Table 3, and Fig. 4 displays the DVH curves. The 95% CI for the means using the standard error of the mean is: $\overline{D}_{90}$ = [16.27, 16.36] which was significant compared with 16 Gy (p value < 0.001); and the percentile 95% CI for *V*_100_ = [92.55, 93.50] was significantly above the 90% volume threshold (p value < 0.001). For hotspots in the prostate, $\overline{V}_{150}$ = [23.61, 24.05] was significantly below the constraint (p value < 0.001) and $\overline{V}_{200}$ = [10.29, 10.46] was significantly above the 10% volume constraint (p value < 0.001). These results indicate the prostate is likely to receive a dose distribution that covers 90% of the volume with the prescribed dose with only $\overline{V}_{150}$ within constraints, with respect to the 95% confidence level. The 95% CI for urethral constraints were $\overline{D}_{10}$ =  [17.19, 17.30] and $\overline{D}_{{0.01{\text{ cc}}}}$ =  [17.77, 17.91], and both were significantly below the dose constraints (p values < 0.001). For the rectum, the percentile 95% CI for *V*_75_ = [0.13, 0.18] and was significantly below the 1.0 cc constraint (p-values < 0.001), as was $\overline{D}_{{0.1{\text{ cc}}}}$ = [12.16, 12.26]. This shows there is a high likelihood of clinical objectives being met for both the urethra and rectum.

**Table 3:** DVH metric summary data from the robust evaluation of an HDR prostate brachytherapy patient treatment plan

	Dose-volume metrics	Nominal dose-volume metrics	Probabilistic (mean ± SD)	Worst-case	No. passed for probabilistic (Total: 1000)
*Prostate*	*D*_90_ (Gy) > 16	16.5	16.31 ± 0.73	Min = 12.02	641 (64.1%)
	*V*_100_ (%) > 90	94.6	Med = 93.1 Q1 = 87.4 Q3 = 96.5	Min = 28.0	641 (64.1%)
	*V*_150_ (%) < 40	24.1	23.8 ± 3.5	Max = 72.2	1000 (100.0%)
	*V*_200_ (%) < 10	10.7	10.4 ± 1.3	Max = 24.6	405 (40.5%)
*Urethra*	*D*_10_ (Gy) < 18.4	17.1	17.25 ± 0.89	Max = 23.7	892 (89.2%)
	*D*_0.01 cc_ (Gy) < 18.4	17.3	17.8 ± 1.2	Max = 25.4	739 (73.9%)
*Rectum*	*V*_75_ (cc) < 1.0	0.12	Med = 0.16 Q1 = 0.03 Q3 = 0.45	Max = 5.22	946 (94.6%)
	*D*_0.1 cc_ (Gy) < 13	12.0	12.21 ± 0.79	Max = 21.76	841 (84.1%)

The median (Med) and first and third quartile (Q1 and Q3) values are given for the prostate *V*_100_ and rectum *V*_75_ metrics in the probabilistic uncertainty scenarios since these distributions differed from a normal distribution. The maximum (Max) or minimum (Min) are given for the worst-case uncertainty scenarios

Worst-case to the prostate for this TP involved dwell points moving superior along the needle (2.47 mm) and out from the prostate centre (2.47 mm); dwell times decreasing (7.2%); and enlargement of the prostate volume (1.65 mm from centre), shown in Fig. 4a as the furthest left DVH curve, and resulted in *D*_90_ of 11.87 Gy. The dose metric *V*_100_ had the worst value of 26.72% coverage of the prostate volume and resulted from the same TP changes as mentioned with two exceptions, the prostate contour was contracted (1.65 mm towards centre), and the dwell points moved inferiorly (2.47 mm). With respect to the prostate worst-case resulting in the highest *V*_200_ (18.0%), the opposite TP changes to *D*_90_ worst-case were observed except with no SI direction change to dwell points. For the urethra, a *D*_10_ of 23.7 Gy was the worst-case resulting from the urethra expanding (0.41 mm), dwell points moving superior (2.47 mm) and inwards to the prostate centre line (2.47 mm), and dwell times increasing (7.2%). The worst-case value for the rectum occurred with a *V*_75_ of 5.22 cc, equating to dwell points moving superior (2.47 mm) and posteriorly (0.82 mm), dwell times increasing (7.2%), and no change to the rectum boundary.

## Discussion

**Fig. 5:**
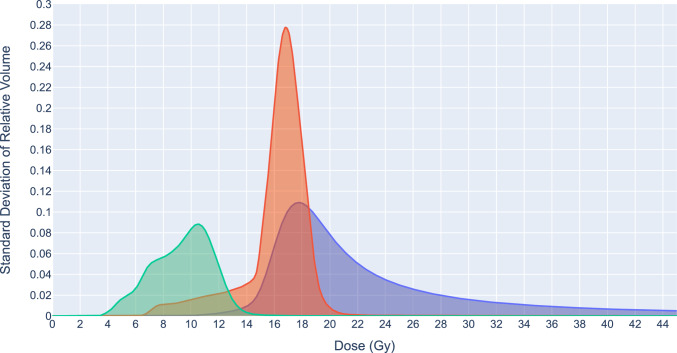
The distribution of standard deviations of relative volumes from the probabilistic robust evaluation method per dose. The area quantifies the total variation in dose across the volume of the structure. The blue area is the prostate (0.95 Gy), the red area is the urethra (0.91 Gy), and the green area is the rectum (0.45 Gy)

The robustness of a TP against uncertainties can be quantified by the sensitivity of a nominal plan’s DVH data to changes in the plan parameters. This sensitivity is quantified by the standard deviation (or first and third quartiles) in the DVH metrics for each organ in the probabilistic scenarios, Table 3. Although the DVH metrics are clinically important, they quantify robustness at single points along the DVH curve. A more informative approach to quantify the robustness of the nominal plan is the sum of standard deviations per dose, which gives a measure of variation along the entire DVH curve, shown in Fig. 5. This allows a comparison of robustness between structures with the rectum the most robust to uncertainties, while the urethra and prostate were approximately double in area, indicating a much higher sensitivity to uncertainties. A judgment of the significance of these results needs to be correlated with retrospective patient outcomes for a cohort of previous patient plans to assess the nominal plans’ robustness in context [24]. A measure of plan quality is seen in the pass-rate of DVH metrics in the probabilistic scenarios. The pass-rate of DVH metrics shown in Table 3 also needs to be benchmarked against the historical record. Providing this historical data is planned in future research by expanding the patient number in the study.

The worst-case scenarios deviate considerably from the nominal case and are therefore concerning when considering the clinical implications. For one of these scenarios to be realised all uncertainties need to be an extreme value, which carries an extremely low non-zero probability. Considering just the 16 needles all moving superiorly and out from the prostate centre line, a total of 32 random movements are involved with in the algorithm. The probability that any one of these uncertainties occur at the most extreme value used in the algorithm (± 1.65 SD) or more extreme is 0.05, and for all 32 to be extreme at the same time is approximately zero. More probable is uncertainties in an unfavourable direction will be compensated by uncertainties in more favourable directions which can be seen by the 1000 probabilistic scenarios being clustered around the nominal DVH curve in Fig. 4. The utility of the worst-case scenarios lies in their use as a point of comparison between competing plans for the same patient, comparison against a historical set of plans, and their inclusion as a point of optimisation in robust optimisation, which is future work.

Algorithm runtime is of clinical significance. The dose calculation is the major contributor to computation time, and this might be solved by GPU processing and limiting the number of dose calculation points. Bouter et al. [48] calculated dose at 100 000 points in multiple plans in 3 minutes using an NVIDIA Titan Xp GPU. The algorithm presented in this study is not written explicitly to utilize multiprocessing; however, an attempt was made to vectorize all processes and take advantage of python modules [47, 49] which natively utilize multithreading.

Further time saving will be achieved by limiting the number of scenarios. In future work, a larger patient cohort will be evaluated with this framework, reducing the number of worst-case scenarios needed by highlighting the common movements resulting in the worst-case scenarios. The number of probabilistic scenarios could be limited by considering an acceptable standard error of the mean for each DVH metric. For 30 scenarios, the 95%CI for the mean for *D*_90_ is ± 0.78 Gy; *D*_10_ is ± 0.10 Gy; *V*_75_ is ± 0.034%. These ranges would provide additional information about the plan than is currently available for evaluation and in a clinically acceptable timeframe, particularly if newer hardware and algorithm optimization in the dose calculation were also incorporated.

The created dose calculation within the robust evaluation algorithm had comparable values to the TPS dose calculation with nominal DVH curves having a mean volume difference of 0.02 ± 0.21% (1 SD) at the same dose values. The maximum differences were between the rectum DVH curves at high doses as the curves approached a percentage volume of zero (max diff = 1.6%). DVH metrics between TPS and those calculated by the robust evaluation algorithm where within 1% for the nominal plan, except for the V_75_ metric for the rectum which was 0.16 cc for the TPS dose calculation and 0.12 cc for the algorithms dose calculation. The difference was due to the deviation between the TPS’s and algorithm’s DVH curves for the rectum as the percentage of the volume approached zero.

The selection of uncertainty ranges and types is based on our technique and represents an example of robust evaluation’s implementation, which can be expanded to other techniques. The uncertainty ranges incorporated into the algorithm have a limitation by not obtaining data within our clinic. For example, contouring uncertainties depend on the planner’s experience and established procedures at the clinic. Uncertainties were treated as if their distribution could be approximated by a normal distribution which is most often the case. Kirisits et al. [17] point out that large values (with low probabilities) from sampling a normal distribution may cause an unrealistic change due to the anatomy limiting possible movements. The values chosen considered this and simulated within the 90% CI.

Oedema of organs was not considered due to the timeframe in which treatment occurs and since the ultrasound image was obtained after needle insertion. Rylander et al. [3] concluded that prostate swelling was minimal in live TRUS planned HDR prostate BT since images obtained before and after the procedure had minimal prostate width and height differences. Oedema could be incorporated into the algorithm by increasing the normal distribution SD for variable parameters two and three by the appropriate amount. Another uncertainty not considered was transit dose which isn’t always accounted for accurately by the afterfloader for needles that contain a high proportion of small dwell times [19]. Including transit dose was beyond the scope of the current study; however, its conclusion into the algorithm is possible by increasing the dwell times in variable parameter five accordingly.

Approximations in the simulation of uncertainties were made to strike a balance between algorithm performance and realistic changes. The prostate boundary is changed in all directions by the same magnitude; however, from Table 2, the prostate length uncertainty is approximately double that of the other two dimensions. For algorithm speed, it was more beneficial to expand from one point with the same magnitude, and it is assumed that the chosen magnitude balances the directional differences, producing the same sized volume as if three different magnitudes were applied per perpendicular direction. A further approximation was within the dose calculation, with the change in orientation of the source assumed not to change when dwell points were moved within the transverse slice. It is also documented that the uncertainty of needle path reconstruction in the transverse plane increases with distance from the TRUS probe; Peikeri et al. [44] found differences from 0.5 to 4 mm. This potentially reduces the worst-case to the rectum as the most posterior needles have reduced uncertainty in their location. For algorithm performance, all transverse movements of the dwell points were sampled from the same probability distribution; however, at a speed cost, the movements can be restricted by a gradient radially out from the TRUS probe.

The worst-case scenarios involved assumptions of what constitutes the worst-case movements. Scenarios for the worst-case were sampled from 90% CI limits for each variable parameter (± 1.65 SD). It was assumed that this would produce the worst-case for each structure; however, this may not be the case, and some combination of values between these limits may result in a more extreme worst-case. However, it was observed that no probabilistic DVH was further from the nominal DVH compared to the most extreme worst-case DVH. For dwell points in the transverse plane, it was assumed that the worst-case for the prostate and urethra would involve moving all needles out from or into the prostate centreline. Needle shifts from the urethra centreline were not simulated but could result in a worst-case for the urethra. Also, the needles moving posteriorly toward the rectum were assumed to produce the worst-case for the rectum; however, a radial movement towards the centre of the rectum, per slice, may constitute a worse scenario due to the curvature of the rectum. The impact of these assumptions on the assessed worst-case is a matter of further research.

Applying a margin around the clinical target volume (CTV) to form a planning target volume (PTV) has been a common method of dealing with uncertainties within radiotherapy but is not part of all HDR prostate BT techniques. The robust evaluation algorithm presented could be used to investigate appropriate margins around the CTV using the 3D dose distributions resulting from uncertainty scenarios. The benefit of this method is any margin derived would be clinic specific, using the clinics own uncertainty values and uncertainty types appropriate for their technique. The study would also require a large patient cohort and attention to the impact on OAR.

Dwell time objective smoothing rules have not been investigated in this study. No maximum dwell time was applied to the nominal treatment plan; however, this would impact in the robustness of the nominal treatment plan. Future work will investigate the impact of utilizing dwell time objective smoothing rules to generate more robust treatment plans.

Future research is also planned on incorporating the robust evaluation algorithm into the plan optimization stage. This will provide robustly optimized TP to the combined uncertainties in HDR prostate BT. To date, robust optimization techniques have been limited to one uncertainty type [31, 32] limiting the true impact of uncertainties on the dose distribution delivered to the patient.

## Conclusion

A robust evaluation algorithm was presented and demonstrated on one patient TP in HDR prostate BT. A mean time of 4.43 ± 0.67 s was achieved per scenario. For the patient, the most likely outcome of each DVH metric from the probabilistic scenarios was a dose distribution which covered all clinical objectives, except *V*_*200*_. However, only 7.5% of these scenarios passed all clinical objectives in the same uncertainty scenario; this increases to 43.2% of scenarios passing all clinical objectives when excluding the *V*_*200*_ DVH metric. Regarding robustness, *D*_90_ had a standard deviation of 0.73 Gy and a D_10_ for the urethra of 1.1 Gy. The rectum had a considerable variation in *V*_75_ with an inter-quartile range of 0.42 cc compared to a nominal value for *V*_75_ of 0.12 cc; however, the rectum had the lowest overall sensitivity to uncertainty when considering the dose to the entire volume with the sum of standard deviation for the whole DVH curve approximately half of the value of the prostate or urethra. The worst cases to the prostate results in a decrease of 27.2% in *D*_90_, an increase of 46.8% in the maximum dose to the urethra, and an 81.3% increase in the maximum dose to the rectum. The robust evaluation outcomes calculated for the TP need to be compared against clinical data with historical patient outcomes to assess the results’ significance and hence quantify the TP’s robustness to uncertainties.
